# A Comparison of Three Electrophysiological Methods for the Assessment of Disease Status in a Mild Spinal Muscular Atrophy Mouse Model

**DOI:** 10.1371/journal.pone.0111428

**Published:** 2014-10-27

**Authors:** Jia Li, Tom R. Geisbush, William D. Arnold, Glenn D. Rosen, Phillip G. Zaworski, Seward B. Rutkove

**Affiliations:** 1 Department of Neurology, Beth Israel Deaconess Medical Center, Harvard Medical School, Boston, Massachusetts, United States of America; 2 Department of Neurology, Ohio State University, Columbus, Ohio, United States of America; 3 PharmOptima, LLC, Portage, Michigan, United States of America; University of Edinburgh, United Kingdom

## Abstract

**Objectives:**

There is a need for better, noninvasive quantitative biomarkers for assessing the rate of progression and possible response to therapy in spinal muscular atrophy (SMA). In this study, we compared three electrophysiological measures: compound muscle action potential (CMAP) amplitude, motor unit number estimate (MUNE), and electrical impedance myography (EIM) 50 kHz phase values in a mild mouse model of spinal muscular atrophy, the Smn1^c/c^ mouse.

**Methods:**

Smn1^c/c^ mice (N = 11) and wild type (WT) animals (−/−, N = 13) were measured on average triweekly until approximately 1 year of age. Measurements included CMAP, EIM, and MUNE of the gastrocnemius muscle as well as weight and front paw grip strength. At the time of sacrifice at one year, additional analyses were performed on the animals including serum survival motor neuron (SMN) protein levels and muscle fiber size.

**Results:**

Both EIM 50 kHz phase and CMAP showed strong differences between WT and SMA animals (repeated measures 2-way ANOVA, P<0.0001 for both) whereas MUNE did not. Both body weight and EIM showed differences in the trajectory over time (p<0.001 and p = 0.005, respectively). At the time of sacrifice at one year, EIM values correlated to motor neuron counts in the spinal cord and SMN levels across both groups of animals (r = 0.41, p = 0.047 and r = 0.57, p  = 0.003, respectively), while CMAP did not. Motor neuron number in Smn1^c/c^ mice was not significantly reduced compared to WT animals.

**Conclusions:**

EIM appears sensitive to muscle status in this mild animal model of SMA. The lack of a reduction in MUNE or motor neuron number but reduced EIM and CMAP values support that much of the pathology in these animals is distal to the cell body, likely at the neuromuscular junction or the muscle itself.

## Introduction

A variety of mouse models of spinal muscular atrophy (SMA) have been developed over the past decade [1]. These include animals with relatively severe phenotypes, such as the survival motor neuron negative (Smn^−/−^), Smn2^+/+^, and SMNΔ7 models [2]–[4], all of which die within the first 3 weeks of life, to relatively long lived models, including the Smn1^c/c^ mouse, which develops only subtle motor deficits [5]. The impetus underlying the development of these various models is based on an effort to recapitulate the marked varying human disease severities, with the SMNΔ7 model mimicking Type 1 SMA and Smn1^c/c^ mimicking SMA Type 3. While each of these disease models has its unique features, nearly all of the longer-lived models develop some element of digital, ear, and tail necrosis [5].

A major purpose of these models is to evaluate potential therapies. Beyond measuring survival, which is effective in the study of more severe models [6], [7], biomarkers are needed to evaluate more subtle therapeutic effects, such as disease stabilization or slowing of progression. A variety of such biomarkers have been explored in both animals and humans, including serological biomarkers measures such as SMN expression [8], functional measures [9], muscle imaging [10], [11], as well as electrophysiologic markers, including motor unit number estimate (MUNE) and compound motor action potential amplitude (CMAP) [12]–[14]. More recently, the technique of electrical impedance myography (EIM) has also been studied in this disease. EIM is a bioimpedance-based procedure in which a high-frequency electrical current is applied to a localized area of muscle and the consequent voltages measured [15]. The voltages reflect changes in both muscle membrane health and size and well as local compositional changes to the muscle. A longitudinal study in children with mainly older SMA Type 2 and Type 3 patients has shown the relative stability of EIM values over time, in contrast to normal children who show increasing EIM phase values with age, consistent with progressive muscle growth and maturation [16]. Since EIM is non-invasive, painless, and simple to apply, it may be especially useful in young children and is currently being investigated as a part of a multicenter study.

In an effort to further refine and study EIM as a potential technology that can be readily applied for assessment of SMA therapies in both animals and humans, we undertook a study of the Smn1^c/c^ mouse with two main goals. First, we were interested in identifying differences in EIM, CMAP, and MUNE in a group of Smn1^c/c^ mice longitudinally over an extended period of time (up to 1 year of age). Second, we sought to determine how these measures correlated to serologic biomarkers, including SMN levels and histological data.

## Methods

### Animals

All procedures were approved by the Beth Israel Deaconess Medical Center Institutional Animal Care and Use Committee (IACUC). Breeding colonies of Smn1^c/c^ mice (strain: B6.129-*Smn1^tm5(Smn1/SMN2)Mrph^*/J) were established from animals obtained from Jackson Labs (Bangor, Maine). Animals were genotyped by tail snip.

Given that this animal model develops necrosis of the tail, pinna of ear, and digits of hind-paw up to an age of approximately P90, special care was mandated by our animal research facility. Specifically, animals were not weaned until P28. Special bedding and gel-packs (DietGel 76A, PharmaSer, Framingham, MA) were provided at all times. As long as necrosis was present, the animal was provided meloxicam subcutaneously daily. Any animal developing paw (as compared to digital necrosis) or losing more than 20% body weight was euthanized. No electrophysiological or behavioral measurements were allowed until the necrosis had completely resolved, which was at approximately 15 weeks of age. A total of only 7 Smn1^c/c^ and 7 wild-type (WT) mice were ultimately studied for the full 15–52 week duration post necrosis and included in the longitudinal analysis. An additional 4 Smn1^c/c^ and corresponding 6 WT animals were ultimately also included and followed out to 52 weeks, at which time all animals were measured a final time and sacrificed.

### Experimental design

There were two separate components to the study, the first being a longitudinal element, in which animals were followed at a regular intervals from 15 weeks of age until 1 year of age, and the second being a cross-sectional element, when the animals were all sacrificed at approximately 1 year of age. Body weight, front paw grip strength, CMAP, MUNE, and EIM were obtained on a regular basis for 37 weeks. At approximately 1 year of age all animals were sacrificed, at which time gastrocnemius muscle, spinal cord (L4–5), and whole blood were collected for further analysis.

### Functional study

The front paw grip strength was measured by a grip strength meter single computerized sensor with standard pull bars (CAT # 1027CSM, Columbus Instruments). The animal was allowed to grasp a small bar connected to a sensitive force transducer. Holding the lower back of the animal, the investigator (JL) pulled the animal away from the bar until it lost its grip. The maximum force recorded out of 5 trials was recorded.

### CMAP and MUNE measurements

CMAP and MUNE were performed on the left hind limb via TECA Synergy T2 EMG Monitor System (Viasys, Madison, WI) while the animal was under anesthesia, as previously described [17]. Briefly, the sciatic nerve was supramaximally stimulated at the sciatic notch and the entire distal leg muscle compartment recorded via disposable ring electrodes with a ground electrode placed on the right hind paw to record the CMAP [18], [19]. For MUNE, we followed a previously published incremental approach for use in animals described by others [20], [21]. Briefly, 10 incremental steps were recorded and averaged to determine the average single motor unit potential amplitude. The MUNE was calculated by dividing the CMAP amplitude by the average single motor unit potential amplitude. Of note, the MUNE analysis would have been more complete had multiple methods been used to confirm the MUNE, including multipoint [22] and modified multipoint [23]. However, these were not attempted given the already complex data set being collected; in addition, performing these methods in a mouse are extremely difficult given their small size and the need to move stimulating electrodes to different point along the nerve.

### EIM study

All EIM measurements were performed with the animals placed under 1% isoflurane anesthesia delivered through a nose cone with a heating pad underneath the limb to maintain consistent temperature. The leg was then taped to the measuring surface at an approximately 45° angle extending out from the body, away from the head.

A fixed 4-electrode array was placed over the gastrocnemius muscle, as previously described [17]. EIM measurements were performed with a Skulpt Inc EIM1103 system (San Francisco, CA).

### Serological, pathological and histological studies

1. SMN protein concentration. The whole blood was obtained via cardio-puncture and preserved at −80°C. These samples were analyzed by PharmOptima with an electrochemiluminescence-based SMN immunoassay developed by PharmOptima (Portage, MI), using Meso Scale Discovery technology. 2. Muscle weight and myocyte fiber area. The entire gastrocnemius muscle was immediately excised at its proximal end just below the knee and cutting the gastrocnemius tendon distally, and its mass obtained. The muscle was then snap-frozen in isopentane cooled in liquid nitrogen and stored at −80°C. The tissue was then cut into 5 µm slices and stained with hematoxylin and eosin. Stereological measurements were made using a Zeiss Axiophot microscope with a motorized stage interfaced with a Dell Optiflex 380 computer running Stereo Investigator (MBF Biosciences, Inc., Williston, VT) software. Approximately 30 cells from each muscle were evaluated in a blinded fashion by a single investigator (TG). 3. Hydroxyproline level. In order to assess the quantity of connective tissue in the muscle as a consequence of disuse/neuronal loss, a commercially available assay (Kit #6017, Chondrex, Inc. Redmond, WA) was used to measure muscle hydroxyproline content. 4. Spinal cord motor neuron number. All animals were perfused and then fixed with 4% Formalin. To keep the spinal cord intact, the whole spinal column was removed and then decalcified by EDTA over several days. Then, the lumbar 4 and 5 levels of the spinal cord were removed, cut into 5 µm section in paraffin, and stained by cresyl violet. Bilateral motor neuron number in the anterior horn on each section was counted blind to group designation. Regions of interest were counted at 40X magnification using a Zeiss Axiophot microscope interfaced with a Dell Optiflex 380 computer running Stereo Investigator (MF Biosciences, Inc, VT). The region of interest was sub-divided into sections using the optical fractionator within the Stereo Investigator software and examined individually. Inclusion of a cell as a motor neuron was based on cell size, centering of nucleus within the cell, and the amount of cytoplasm within the cell.

### Data analysis

In order to utilize all the data obtained, differences between animals were initially assessed via repeated measures 2-way ANOVA, in which the independent variable was group and dependent variable the individual electrophysiological measures. However, in order to mirror standard practices in clinical trials, trajectories of change were then calculated for each individual animal over time (fit via linear regression anchored to the baseline visit), comparing the slopes of the trajectories for each of the measures between groups of animals. Unpaired t-tests and Pearson correlation analyses were also performed to determine the relationship between various functional, electrophysiological, pathological, and histological studies at the time of sacrifice at one year of age. For all statistical tests, significance was determined at p<0.05, two-tailed. All results are summarized as mean ± standard error.

## Results

### Differences between groups for the three main physiological measures: CMAP, MUNE, and EIM

In order to effectively capture all the data from multiple visits in the comparison between groups, we performed a two-way repeated measures ANOVA. There was a highly significant group effect for EIM (F(12,130)  = 44, p<0.001) and a similarly strongly significant group effect for CMAP (F (12,130) = 26, p<0.001) but a non-significant group effect for MUNE (F (12,130) = 0.31, p = 0.58,). A significant interaction term (p<0.001) was also present for EIM but not MUNE or CMAP.

### Longitudinal trajectories for all measures

**Figure 1:**
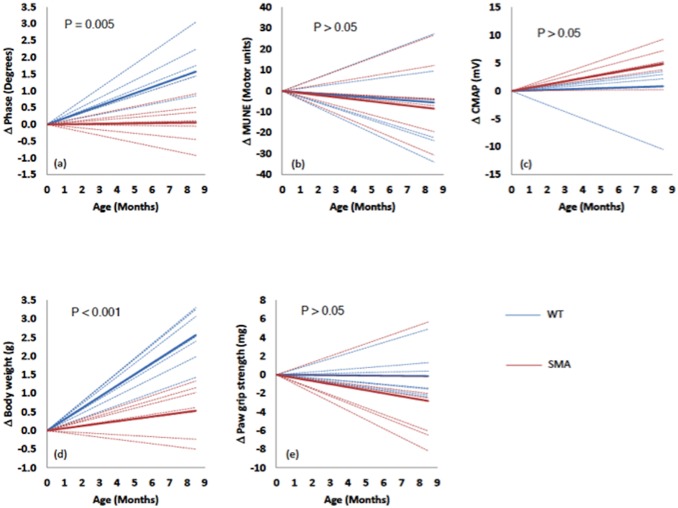
Trajectories of change (± standard deviation) over the period of 15 weeks to 52 weeks for the 7 Smn1^c/c^ animals (in black) and 7 WT animals (in gray) followed for the entire duration. Data is normalized to baseline with a least squares fit of the data. Only weight and EIM showed significant differences in the trajectories between the groups.

As Figure 1 demonstrates, of the three electrophysiological parameters, only EIM demonstrated a significant difference over time in the SMA mice as compared to the WT mice, although the difference appeared to be due mainly to increasing values for the WT animals rather than to a reduction in values in the SMA animals. Animal weight showed a similar trajectory.

### End point-cross sectional study

**Figure 2:**
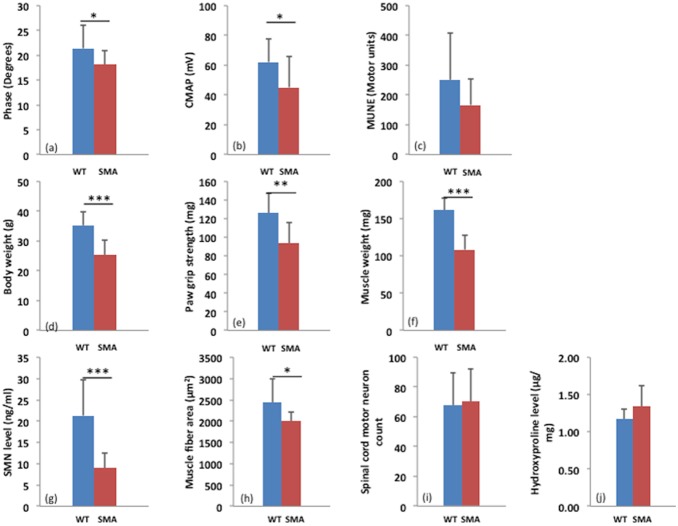
Column plots comparing 1-year end point data for WT and SMA animals. While most of the readily obtained measures, including body weight, paw grip strength, and muscle size are smaller and SMN is markedly reduced, all electrophysiological parameters show only modest differences. In addition, hydroxyproline, a measure of muscle fibrosis, is non-significantly increased in the SMA animals. *<0.05, **<0.01, ***<0.001.

Figure 2 provides a summary of the endpoint data for the two groups of animals. Figure 2a, 2b, and 2c show that body weight, front paw grip strength, and gastrocnemius muscle mass in the SMA animals were reduced compared to controls (p<0.01 for all); of note, muscle mass was 32.8% lower in the SMA animals as compared to WT. Figure 2d, 2e and 2f show that there were borderline significant differences between the groups for both EIM (p = 0.045) and CMAP (p = 0.043); MUNE showed no significant difference between the groups.

Figure 2g, 2h, 2i and 2j showed the significant differences between the groups for both SMN concentration (p<0.001) and myocyte area (p = 0.043); Spinal cord motor neuron count and hydroxyproline level did not show a significant difference between the groups.

**Figure 3:**
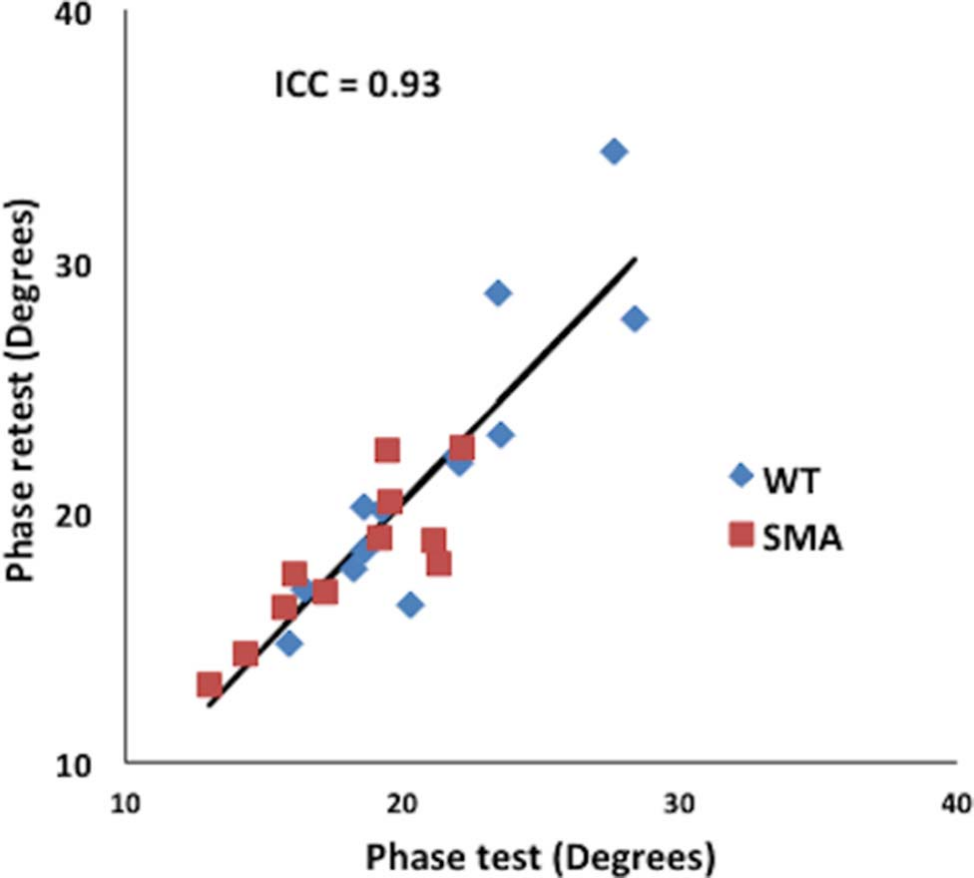
Test-retest reproducibility of EIM recorded from the mouse gastrocnemius at the one year end point.

Figure 3 shows test-retest repeatability of the endpoint data; as can be seen, the repeatability is good with an intra-class correlation coefficient of greater than 0.90.

### End Point Correlations

**Figure 4:**
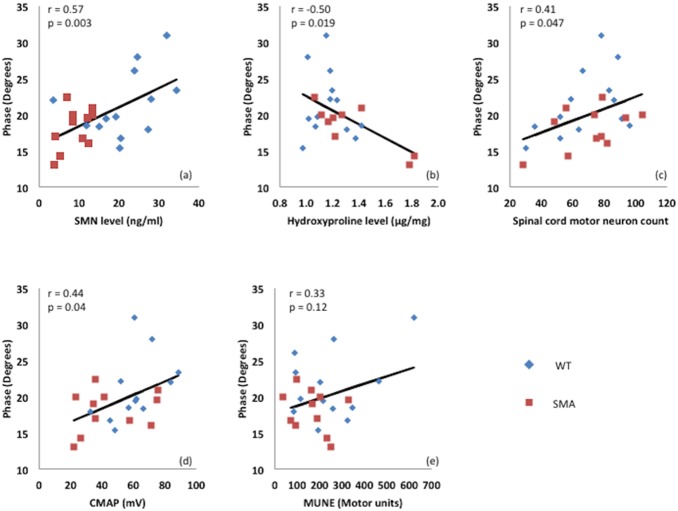
A selection of the correlations between various parameters for both animal types combined.

The correlations are summarized in Table 1, with selected correlations provided in Figure 4. Among electrophysiological measures, only EIM showed the most consistent correlations with serological, pathological and histological measures, as presented in Figure 4a (SMN concentration, r = 0.57, p = 0.003), 4b (hydroxyproline level, r  =  *−*0.50, p = 0.019), and 4c (spinal cord motor neuron count, r = 0.41, p = 0.047), although it did not correlate significantly with muscle fiber size. Interestingly, EIM phase also correlated to CMAP (Figure 4d, r = 0.44, p = 0.04) whereas it had a poor correlation with MUNE (Figure 4e, r  =  *−*0.33, p = 0.12).

**Table 1:** Summary of correlations.

		Muscle weight	SMN	Hydroxy-proline	Musclefiber area	Motor neuroncount	CMAP	MUNE
SMN	r	0.62						
	p	0.00						
Hydroxy-proline	r	−0.41	−0.37					
	p	0.06	0.09					
Musclefiber area	r	0.39	0.01	0.06				
	p	0.07	0.95	0.78				
Spinal cordmotor	r	−0.05	0.03	−0.22	0.13			
neuroncount	p	0.84	0.89	0.33	0.55			
CMAP	r	0.41	0.39	−0.36	0.36	0.34		
	p	0.08	0.07	0.12	0.12	0.13		
MUNE	r	0.11	0.29	0.09	0.16	0.07	0.13	
	p	0.62	0.17	0.68	0.47	0.76	0.55	
Phase	r	0.26	0.57	−0.50	0.07	0.41	0.44	−0.33
	p	0.25	0.00	0.02	0.75	0.05	0.04	0.12

## Discussion

Overall, the results of this study support that EIM is sensitive to disease status in Smn1^c/c^ mice. Of the 3 electrophysiological biomarkers, both EIM and CMAP revealed significant differences between WT and SMA animals and only EIM revealed a difference in trajectory between the groups over the approximately 36-week period of study. This finding is analogous to the data obtained in our longitudinal study of older children with SMA [16]. While this EIM finding may appear useful, it is important to also point out that body weight showed a very similar trend (compare Figures 1a and 1c), which itself has been suggested as a useful measure in the SMNΔ7 mouse [24]. An obvious concern is that EIM is proving nothing more than a surrogate for body weight. In fact, it is not unreasonable to suspect that the two measures are associated. A major component of body weight is muscle mass, and EIM, while not a direct measure of muscle mass, is deeply impacted by the number and size of muscle fibers [25]. Since more, larger fibers imply greater muscle mass and hence greater body weight, it is not surprising that EIM and body weight show similar trends. Another point worth highlighting is that while the final measurements show a significantly lower CMAP for SMA animals than for WT (Figure 2), the WT CMAP actually decreased slightly over time whereas the SMA increased slightly (Figure 1). This apparent paradox is explained by the fact that the CMAP data in the SMA animals was lower throughout the study, and although both groups’ values converged slightly over time, they never approached the point of equivalence.

Interestingly, there were no significant differences between the number of motor neurons in the spinal cords of the diseased mice versus the healthy mice, consistent with previous work showing a normal number of neurons in the L3 and L4 ventral roots [5]. This is in contrast to features of reduced MUNE and normalized CMAP because of collateral reinnervation that have been reported in patients with mild SMA [12]. This discordance between the animal model and the human disease is problematical and brings into question the value of this model. Still we do know that SMN is expressed in muscle as well [26], so it is possible that we are identifying muscle specific effects that may still be relevant to disease pathogenesis even if not primary motor neuron pathology. What remains surprising, however, is that despite this, EIM correlated to the motor neuron counts, perhaps suggesting some general relationship between EIM values and the number of motor neurons in a limb. It is also intriguing that EIM correlated to the SMN protein concentration, a second somewhat unexpected finding. Since SMN protein is expressed in muscle [26], it is possible that SMN levels are correlating closely with muscle size and weight, both of which are being captured in the EIM 50 kHz phase data.

How can these seemingly disparate results be integrated? Since there is no apparent loss of motor neurons, either by MUNE or motor neuron counting in the spinal cord, the mildly reduced CMAP and EIM values in the SMA as compared to WT suggest pathology at the distal motor neuron or in the muscle itself. The substantially greater loss of muscle weight as compared to reduction in fiber size (34% vs. 18%) suggests that actual loss of individual muscle fibers (e.g., due to denervation) rather than just limited atrophy may be playing an important role in these findings. Loss of motor neuromuscular junctional input, with subsequent severe fiber atrophy, would be consistent with evidence that SMN plays an important role in the distal nerve and skeletal muscle [27], [28]. Indeed, other work has shown that neuromuscular junctions are abnormal in this model [5]. Primary muscle abnormalities would have the effect of leaving MUNE unaltered while at the same time reducing the EIM and CMAP values [29], Further supporting this interpretation is the increase in hydroxyproline in the SMA animal muscle, suggesting increased connective tissue in the muscle, consistent with myopathic features that have been described in SMA [30].

A major limitation of this study is that of 220 SMA animals bred, only 11 ultimately could be studied due limitations placed on us by our institution’s animal ethics committee. Inasmuch as the degree of necrosis parallels the degree of motor neuron loss, we may have evaluated only the most mildly affected animals. Second, the small number of animals studied, the large number of analyses performed, and the relatively modest p values for a number of the outcomes implies that the study is subject to both type 1 and type 2 error. Third, we only evaluated gastrocnemius, a mildly affected muscle in this model [31]. However, we have been developing approaches for measuring other muscles in the mouse including tibialis anterior, quadriceps, fore limb and even axial muscles using minute needle electrode arrays. Thus, this work can be viewed as only an initial effort toward assessing muscle condition in SMA mice using EIM. Fourth, we have only focused on a single frequency of electrical current in these studies; it is possible that multifrequency metrics could provide additional valuable data.

In summary, these data support that EIM may a play a useful role in the evaluation of mild SMA animals and that EIM data correlate to some extent with other biomarkers. It also supports its potential application in the study of human SMA. However, it is clear that additional study of this and other models of SMA will be needed to fully understand the complex relationship between electrophysiological biomarkers and disease status.

## References

[1] Sleigh JN, Gillingwater TH, Talbot K (2011) The contribution of mouse models to understanding the pathogenesis of spinal muscular atrophy. Dis Model Mech 4: 457–467. 21708901 10.1242/dmm.007245 PMC3124050

[2] Hsieh-Li HM, Chang JG, Jong YJ, Wu MH, Wang NM, et al (2000) A mouse model for spinal muscular atrophy. Nat Genet 24: 66–70. 10615130 10.1038/71709

[3] Monani UR, Sendtner M, Coovert DD, Parsons DW, Andreassi C, et al (2000) The human centromeric survival motor neuron gene (SMN2) rescues embryonic lethality in Smn(−/−) mice and results in a mouse with spinal muscular atrophy. Hum Mol Genet 9: 333–339. 10655541 10.1093/hmg/9.3.333

[4] Le TT, Pham LT, Butchbach ME, Zhang HL, Monani UR, et al (2005) SMNDelta7, the major product of the centromeric survival motor neuron (SMN2) gene, extends survival in mice with spinal muscular atrophy and associates with full-length SMN. Hum Mol Genet 14: 845–857. 15703193 10.1093/hmg/ddi078

[5] Osborne M, Gomez D, Feng Z, McEwen C, Beltran J, et al (2012) Characterization of behavioral and neuromuscular junction phenotypes in a novel allelic series of SMA mouse models. Hum Mol Genet 21: 4431–4447. 22802075 10.1093/hmg/dds285 PMC3459466

[6] Foust KD, Wang X, McGovern VL, Braun L, Bevan AK, et al (2010) Rescue of the spinal muscular atrophy phenotype in a mouse model by early postnatal delivery of SMN. Nat Biotechnol 28: 271–274. 20190738 10.1038/nbt.1610 PMC2889698

[7] Nizzardo M, Simone C, Salani S, Ruepp MD, Rizzo F, et al.. (2014) Effect of combined systemic and local morpholino treatment on the spinal muscular atrophy delta7 mouse model phenotype. Clin Ther 36: 340–356 e345. 10.1016/j.clinthera.2014.02.004 24636820

[8] Finkel RS, Crawford TO, Swoboda KJ, Kaufmann P, Juhasz P, et al (2012) Candidate proteins, metabolites and transcripts in the Biomarkers for Spinal Muscular Atrophy (BforSMA) clinical study. PLoS One 7: e35462. 22558154 10.1371/journal.pone.0035462 PMC3338723

[9] Chen TH, Yang YH, Mai HH, Liang WC, Wu YC, et al.. (2013) Reliability and Validity of Outcome Measures of In-Hospital and At-Home Visits in a Randomized, Double-Blind, Placebo-Controlled Trial for Spinal Muscular Atrophy. J Child Neurol. 10.1177/0883073813506935 24163397

[10] Sproule DM, Montgomery MJ, Punyanitya M, Shen W, Dashnaw S, et al (2011) Thigh muscle volume measured by magnetic resonance imaging is stable over a 6-month interval in spinal muscular atrophy. J Child Neurol 26: 1252–1259. 21572051 10.1177/0883073811405053

[11] Wu JS, Darras BT, Rutkove SB (2010) Assessing spinal muscular atrophy with quantitative ultrasound. Neurology 75: 526–531. 20697104 10.1212/WNL.0b013e3181eccf8f PMC2918474

[12] Bromberg MB, Swoboda KJ (2002) Motor unit number estimation in infants and children with spinal muscular atrophy. Muscle Nerve 25: 445–447. 11870724 10.1002/mus.10050 PMC4334581

[13] Lewelt A, Krosschell KJ, Scott C, Sakonju A, Kissel JT, et al (2010) Compound muscle action potential and motor function in children with spinal muscular atrophy. Muscle Nerve 42: 703–708. 20737553 10.1002/mus.21838 PMC2964439

[14] Arnold WD, Porensky PN, McGovern VL, Iyer CC, Duque S, et al (2014) Electrophysiological Biomarkers in Spinal Muscular Atrophy: Preclinical Proof of Concept. Ann Clin Transl Neurol 1: 34–44. 24511555 10.1002/acn3.23 PMC3914317

[15] Rutkove SB (2009) Electrical Impedance Myography: Background, Current State, and Future Directions. Muscle Nerve 40: 936–946. 19768754 10.1002/mus.21362 PMC2824130

[16] Rutkove SB, Gregas MC, Darras BT (2012) Electrical impedance myography in spinal muscular atrophy: A longitudinal study. Muscle & nerve 45: 642–647. 22499089 10.1002/mus.23233

[17] Li J, Sung M, Rutkove SB (2013) Electrophysiologic biomarkers for assessing disease progression and the effect of riluzole in SOD1 G93A ALS mice. PLoS One 8: e65976. 23762454 10.1371/journal.pone.0065976 PMC3675066

[18] Yalvac ME, Arnold WD, Hussain SR, Braganza C, Shontz KM, et al (2014) VIP-expressing dendritic cells protect against spontaneous autoimmune peripheral polyneuropathy. Mol Ther 22: 1353–1363. 24762627 10.1038/mt.2014.77 PMC4089012

[19] Xia RH, Yosef N, Ubogu EE (2010) Dorsal caudal tail and sciatic motor nerve conduction studies in adult mice: technical aspects and normative data. Muscle Nerve 41: 850–856. 20151466 10.1002/mus.21588

[20] Shefner JM (2001) Motor unit number estimation in human neurological diseases and animal models. Clin Neurophysiol 112: 955–964. 11377252 10.1016/s1388-2457(01)00520-x

[21] Souayah N, Potian JG, Garcia CC, Krivitskaya N, Boone C, et al (2009) Motor unit number estimate as a predictor of motor dysfunction in an animal model of type 1 diabetes. Am J Physiol Endocrinol Metab 297: E602–608. 19602580 10.1152/ajpendo.00245.2009 PMC2739699

[22] Shefner JM, Cudkowicz ME, Brown RH Jr (2002) Comparison of incremental with multipoint MUNE methods in transgenic ALS mice. Muscle Nerve 25: 39–42. 11754183 10.1002/mus.10000

[23] Kasselman LJ, Shefner JM, Rutkove SB (2009) Motor unit number estimation in the rat tail using a modified multipoint stimulation technique. Muscle Nerve 40: 115–121. 19533644 10.1002/mus.21248 PMC4287455

[24] Robbins KL, Glascock JJ, Osman EY, Miller MR, Lorson CL (2014) Defining the therapeutic window in a severe animal model of Spinal Muscular Atrophy. Hum Mol Genet. 10.1093/hmg/ddu169 PMC4119406 24722206

[25] Ahad MA, Fogerson PM, Rosen GD, Narayanswami P, Rutkove SB (2009) Electrical characteristics of rat skeletal muscle in immaturity, adulthood, and after sciatic nerve injury and their relation to muscle fiber size. Physiol Meas 30: 1415–1427. 19887721 10.1088/0967-3334/30/12/009 PMC2821572

[26] Coovert DD, Le TT, McAndrew PE, Strasswimmer J, Crawford TO, et al (1997) The survival motor neuron protein in spinal muscular atrophy. Hum Mol Genet 6: 1205–1214. 9259265 10.1093/hmg/6.8.1205

[27] Williams BY, Vinnakota S, Sawyer CA, Waldrep JC, Hamilton SL, et al (1999) Differential subcellular localization of the survival motor neuron protein in spinal cord and skeletal muscle. Biochem Biophys Res Commun 254: 10–14. 9920724 10.1006/bbrc.1998.9885

[28] Boyer JG, Murray LM, Scott K, Repentigny YD, Renaud JM, et al (2013) Early onset muscle weakness and disruption of muscle proteins in mouse models of spinal muscular atrophy. Skeletal Muscle 3: 1–13. 24119341 10.1186/2044-5040-3-24 PMC3852932

[29] Paganoni S, Amato A (2013) Electrodiagnostic evaluation of myopathies. Phys Med Rehabil Clin N Am 24: 193–207. 23177039 10.1016/j.pmr.2012.08.017 PMC4435557

[30] Mastaglia FL, Walton JN (1971) Histological and histochemical changes in skeletal muscle from cases of chronic juvenile and early adult spinal muscular atrophy (the Kugelberg-Welander syndrome). J Neurol Sci 12: 15–44. 4250701 10.1016/0022-510x(71)90249-8

[31] Ling KK, Gibbs RM, Feng Z, Ko CP (2012) Severe neuromuscular denervation of clinically relevant muscles in a mouse model of spinal muscular atrophy. Hum Mol Genet 21: 185–195. 21968514 10.1093/hmg/ddr453 PMC3235013

